# Vasodilator effects of dehydroepiandrosterone (DHEA) on fetal pulmonary circulation: An experimental study in pregnant sheep

**DOI:** 10.1371/journal.pone.0198778

**Published:** 2018-06-27

**Authors:** Dyuti Sharma, Hélène Coridon, Estelle Aubry, Ali Houeijeh, Véronique Houfflin-Debarge, Rémi Besson, Philippe Deruelle, Laurent Storme

**Affiliations:** 1 Univ. Lille, EA 4489 – Perinatal Environment and Health, Lille, France; 2 CHU Lille, Department of Pediatric Surgery, Hospital Jeanne de Flandre, Lille, France; 3 Hospital MFME, Department of Pediatric Surgery, Fort-de‐France, Martinique, France; 4 CHU Lille, Department of Neonatology, Hospital Jeanne de Flandre, CHRU Lille, Lille, France; 5 CHU Lille, Department of Obstetrics and Gynecology, Hospital Jeanne de Flandre, Lille, France; The University of Manchester, UNITED KINGDOM

## Abstract

Persistent pulmonary hypertension (PPHN) remains a severe complication of the transition to extra-uterine life with significant morbidity and mortality in the newborns. Dehydroepiandrosterone (DHEA) represents a new pharmacological agent with vascular effects, including improvement of PPHN in several animal models. We hypothesized that DHEA could decrease pulmonary vascular resistance (PVR) in the pulmonary circulation of fetal sheep. We studied the effect of intravenous infusion of DHEA in fetal lambs using chronically instrumented sheep at 128 days of gestation. PVR was computed before and after intravenous infusion of increasing doses of DHEA. We assessed pre-treatment by L-nitroarginine, an inhibitor of NO production. Blood gases and doses of DHEA were measured in both sheep and fetus before/after DHEA infusion. Intravenous infusion of DHEA had a vasodilator effect with a significant decrease in PVR (respectively -11%, -14% and -36% after infusion of 6, 12 and 24 mg DHEA, p<0.01) without damaging effects on systemic circulation or on blood gases. The inhibitory effect of pre-treatment with L-nitroarginine resulted in a significant increase in PVR. We demonstrated a potent vasodilator effect of DHEA on fetal pulmonary circulation without deleterious effects. DHEA might represent a new treatment for PPHN.

## Introduction

Persistent pulmonary hypertension (PPH) is a severe disease of multifactorial etiology that may result in significant hemodynamic changes, such as severe pulmonary vascular remodeling, persistent elevation of pulmonary vascular resistance (PVR), right ventricular failure and even death [1]. Multiple mechanisms contribute to normal circulatory adaptation to extra-uterine life [2–6]. The lack of physiological decrease in PVR in PPHN leads to elevated pulmonary artery pressure, decreases pulmonary blood flow, right-to-left shunting of blood at the level of the foramen ovale and ductus arteriosus with severe hypoxemia and circulatory compromise [7]. Inhaled nitric oxide has reduced the need for extracorporeal membranous oxygenation (ECMO) and improved the prognosis of these children [8]. However, refractory hypoxemia may persist, and the search for other therapeutic alternatives is necessary.

Dehydroepiandrosterone (DHEA) and its 3 β-sulfate ester (DHEA-S), which is a steroid hormone secreted by the adrenal cortex, are the most abundant circulating steroid hormone in humans [9]. However, blood levels of DHEA gradually decrease with advancing age [10,11]. A similar pattern was observed for DHEA-S during pregnancy in which the fetal concentration of DHEA-S decreased linearly with gestational age [12].

Many studies have suggested that DHEA has cardiovascular protective properties and many other beneficial effects (“anti-aging hormone”) [13,14].

Although the role of DHEA in humans is not clearly defined, low concentration of DHEA has been associated with in the susceptibility to or development of cardiovascular disease or pulmonary hypertension [15,16]. Recent studies have focused on DHEA activity in the pulmonary circulation. DHEA improves chronic pulmonary hypertension in rodents (induced by chronic hypoxia or monocrotaline injection) [17–21]. In vitro, DHEA causes vasodilation of smooth muscle cells removed from ferret lungs and cultured explants of human pulmonary arteries [22,23]. However, the effect of DHEA on the fetal pulmonary circulation is unknown.

We hypothesized that DHEA could have pulmonary vasodilatory effects in the fetus, which might indicate a therapeutic use for DHEA in PPHN. To test this hypothesis, and to understand the potential mechanisms of action of DHEA, we studied the effect of a bolus infusion of DHEA, both with and without pre-treatment by L-nitroarginine (LNA, inhibitor of NO synthase), on fetal pulmonary circulation in utero using chronically instrumented pregnant sheep.

## Materials and methods

### Animals

The French “Ministère de l’Agriculture, de la Pêche et de l’Alimentation” approved the animal procedures and protocols before the studies were carried out in the Department of Experimental Research at Lille University (animal experimentation agreement n°59286). Pregnant ewes of the Colombia-Rambouillet breed were hosted in individual pens starting a week before (at 120 days of gestational; total gestation duration: 145 days, equivalent to 40 weeks of gestation) and throughout the procedure. Only singletons pregnancies were included in this study.

### Surgical procedures

Fetal chronic instrumentation was performed through an *in utero* surgery at a gestational age between 126 and 128 days (corresponding to 36 weeks of gestation), after fasting for 24 hours. General anesthesia and surgical protocols used for this study were the same as described in previous work [24]. In summary, after a midline laparotomy and hysterotomy in pregnant ewe and after fetal analgesia (10 mg IM nalbuphine) and fetal local anesthesia (50 mg SC lidocaine hydrochloride), catheters (18 G, Vygon, ecoven, France) were positioned in fetal aorta and superior vena cava (SVC) (through axillar vessels). Then after a left thoracotomy, catheters in main pulmonary artery (MPA) and left pulmonary artery (LPA, 21 G catheter) and an ultrasonic flowmeter probe (Transonic^®^, size 6 S, Ithaca, NY, US) around LPA were placed. At the end of hysterotomy closure, an amniotic infusion of 250 mL of isotonic saline serum containing 500 mg of amoxicillin + clavulanic acid (Augmentin Intravenous, 1 g/200 mg, GlaxoSmithKline, Barnard Castle, UK) was carried out through a 16 G catheter left in intra-amniotic cavity to obtain a reference pressure. Postoperative analgesia of ewes was provided by 20 mg IV of nalbuphine that was repeated 6 hours after the first injection and then on a daily basis until the third day after surgery. Similarly, fetal antibiotic prophylaxis (with 500 mg amoxicillin +clavulanic acid through intra-amniotic catheter) and fetal analgesia (10 mg IV nalbuphine) were administered daily until the third day after surgery.

Catheters were maintained by daily 2 mL injections of heparinized (10 IU / mL) normal saline, and their positions were verified at autopsy. In vivo studies were performed after a recovery time of 48 hours to obtain hemodynamic stability.

At the end of the experimental procedures, animals were euthanized using T61^®^ (Tanax^®^, Intervet, Beaucouzé, France) IV infusion (3 mL for 10 kg body weight for the ewe and 0.3 mL/kg for the fetus).

### Physiological measurements and biological analysis

Aortic, MPA and intra-amniotic catheters were registered using a pressure-monitoring system (Merlin, Hewlett-Packard, Palo Alto, CA, USA). Aortic pressure (PAo) and pulmonary arterial pressure (PAP) were referenced to the intra-amniotic pressure (IAP). Heart rate (HR) was calculated using the phasic signal from the PAP.

The LPA flow rate was continuously measured by the flowmeter using the mean of the phasic blood flow signal, with zero blood flow defined as the flow value measured immediately before the beginning of systole. PVR was calculated using the following formula: (PAP—LAP)/LPA flow, where LAP (Left pulmonary Artery Pressure) was previously determined to be equal to IAP+2 [25].

Blood samples (2 mL) from the fetal aortic catheter and from maternal jugular venous catheter were used for blood gas analysis (i-STAT analyzer, Abbott Laboratories, Abbott Park, IL, USA), for oxygen saturation measurement (OSM 3 hemoximeter and ABL 520 Radiometer, Copenhagen, Denmark) and for plasma concentration of DHEA and DHEA-S (dehydroepiandrosterone sulfate) analysis (performed by Radio-Immuno-Assay for DHEA (ng/mL) and by Chemi-Luminescent-Immuno-Assay for DHEA-S (μM/L)).

### Experimental design

Prior to in vivo experimentation, a stable baseline was obtained with saline infusion (6 mL/h) for 30 min through the SVC catheter. Then, the pulmonary hemodynamic response was studied during the 5-min infusion of DHEA (5 mL) or control solution (5 mL) into the SVC followed by saline infusion (5 mL). HR, mean PAP, mean PAo, mean IAP and mean LPA flow were recorded at 10-min intervals before delivering the bolus of DHEA and at 5-min intervals during a 60-min period thereafter. Blood gas and oxygen saturation measurements, as well as blood concentration of DHEA and DHEA-S, were determined for both the ewe and fetus during the baseline period (30 min before DHEA 5-min infusion) and during the 35 min or 65 min (30 min after of DHEA bolus infusion) in each of the following protocols:

- Expt. 1: The effect on fetal pulmonary circulation with increasing doses of DHEA (bolus infusion of 6 mg, 12 mg and 24 mg of DHEA)
  After a stable hemodynamic period of 30 min (baseline period), 6, 12 or 24 mg of DHEA was infused into the fetus as a 5 mL- infusion with saline solution.
  The vascular response to the DHEA infusion was compared to that for the control solution. The control solution was a 5-mL infusion of saline solution at either 2% (6 and 12 mg doses of DHEA) or 4% (24 mg dose of DHEA).
- Expt. 2: The effect on fetal pulmonary circulation with a 5-min infusion of 12 mg and 24 mg of DHEA following an infusion of LNA (inhibition of NO production pathway)
  After a stable hemodynamic period of 30 min (baseline period), 2.4 ml of LNA solution (30 mg) was infused as a bolus into the fetus at time 0 (T0) and was followed by a bolus infusion of 12 or 24 mg of DHEA solution at time T30.
  The vascular response to DHEA after pre-treatment with 30 mg LNA was compared to the vascular response to 30 mg LNA infusion alone.

### Drug preparation

The solutions containing 6 and 12 mg of DHEA (Expt. 1) were prepared using the following procedure: powder of DHEA (prasterone 25 g, Cooper^®^, Melun, France), a liposoluble hormone, was diluted in 2% dimethyl sulfoxide (0.1 mL of DMSO, Sigma-Aldrich Laboratories, St Quentin-Fallavier, France) with addition of 2% Cremophor^®^ 0.1 mL (Sigma-Aldrich Laboratories, St Quentin-Fallavier, France) and 4.8 mL of saline solution.

The solution containing 24 mg of DHEA (Expt. 1) was prepared using the following procedure: powder of DHEA was diluted in 4% dimethyl sulfoxide (0.2 mL) with the addition of 4% Cremophor^®^ 0.2 mL and 4.6 mL of saline solution.

After a solution was prepared, the mixture was buffered with hydrochloric acid and sodium hydroxide to obtain a physiological pH.

The 2% and 4% control solutions (Expt. 1) were prepared, respectively, with 0.1 mL or 0.2 mL DMSO + 0.1 mL or 0.2 mL of Cremophor^®^ in 4.8 mL or 4.6 mL of saline solution.

The LNA solution (Expt. 2) was prepared immediately before in vivo experiments. LNA (30 mg) was dissolved in 0.2 mL of 1 M chlorhydric acid with the addition of 2 mL of normal saline; 0.2 mL of 1 M sodium hydroxide was used to titrate the pH to 7.40.

### Statistical analysis

All statistical analyses were performed using StatView 4.5 software for PC (Abacus Concepts, Berkeley, CA, USA). For both protocols (DHEA and LNA+DHEA), n indicates the number of fetuses studied (independent measures).

Results are expressed as the means ± SEMs or as the percentage of maximum variation from baseline. Intergroup comparisons of the longitudinal changes were performed with an analysis of covariance (ANOVA) and the Bonferroni or Dunn test, using the baseline period (time -30 min to time 0) as the covariate and the treatment period, which included the 60-min period that began after the end of treatment (time 0 to time 65 [Expt. 1] or time 95 [Expt. 2] min), as the repeated observations. Blood gas measurements were compared using the non-parametric Wilcoxon test. A *p* value <0.05 was considered statistically significant. The results were expressed in nmol/L for DHEA (results in ng/mL were converted into nmol/L using reference table [26]) and in μmol/L for DHEA-S.

## Results

A total of 21 singleton pregnant ewes and their fetal lambs (mean weight: 3870 ± 480 g) were used for these experiments (5 groups in Expt. 1 and 2 groups in Expt. 2) with a mean of 2 ± 1 experiments per animal].

### Expt. 1: Vasodilator effect on fetal pulmonary circulation with increasing doses of DHEA 5-min infusion

**Fig 1:**
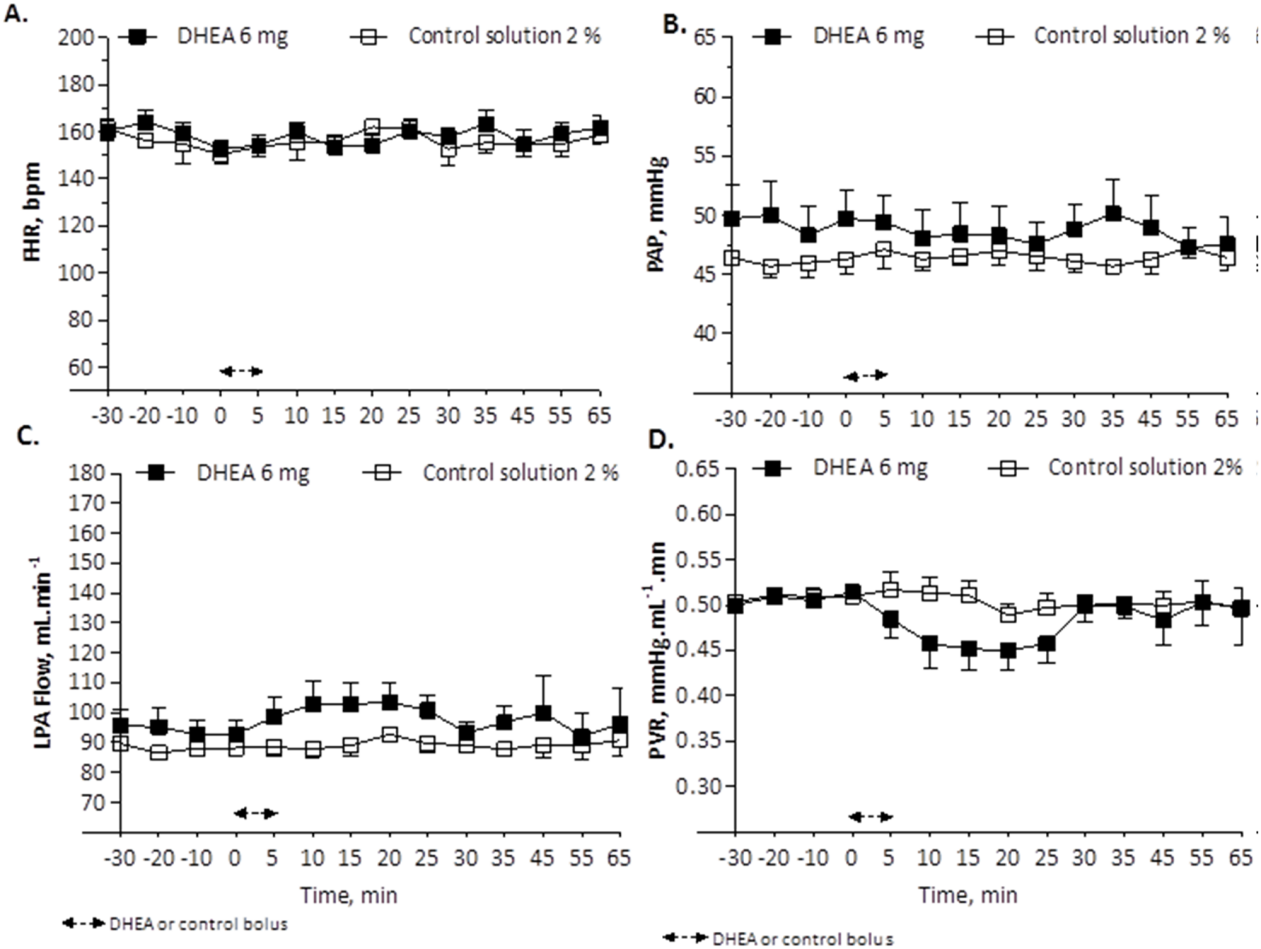
Change in fetal heart rate, FHR (A), pulmonary artery pressure, PAP (B), left pulmonary artery blood flow, LPA flow (C) and pulmonary vascular resistance, PVR (D) in response to bolus of 6 mg DHEA compared to bolus of control solution 2%. Values are means ±SEMs, n = 5, (DHEA 6 mg) and n = 5, (control 2%).

**Fig 2:**
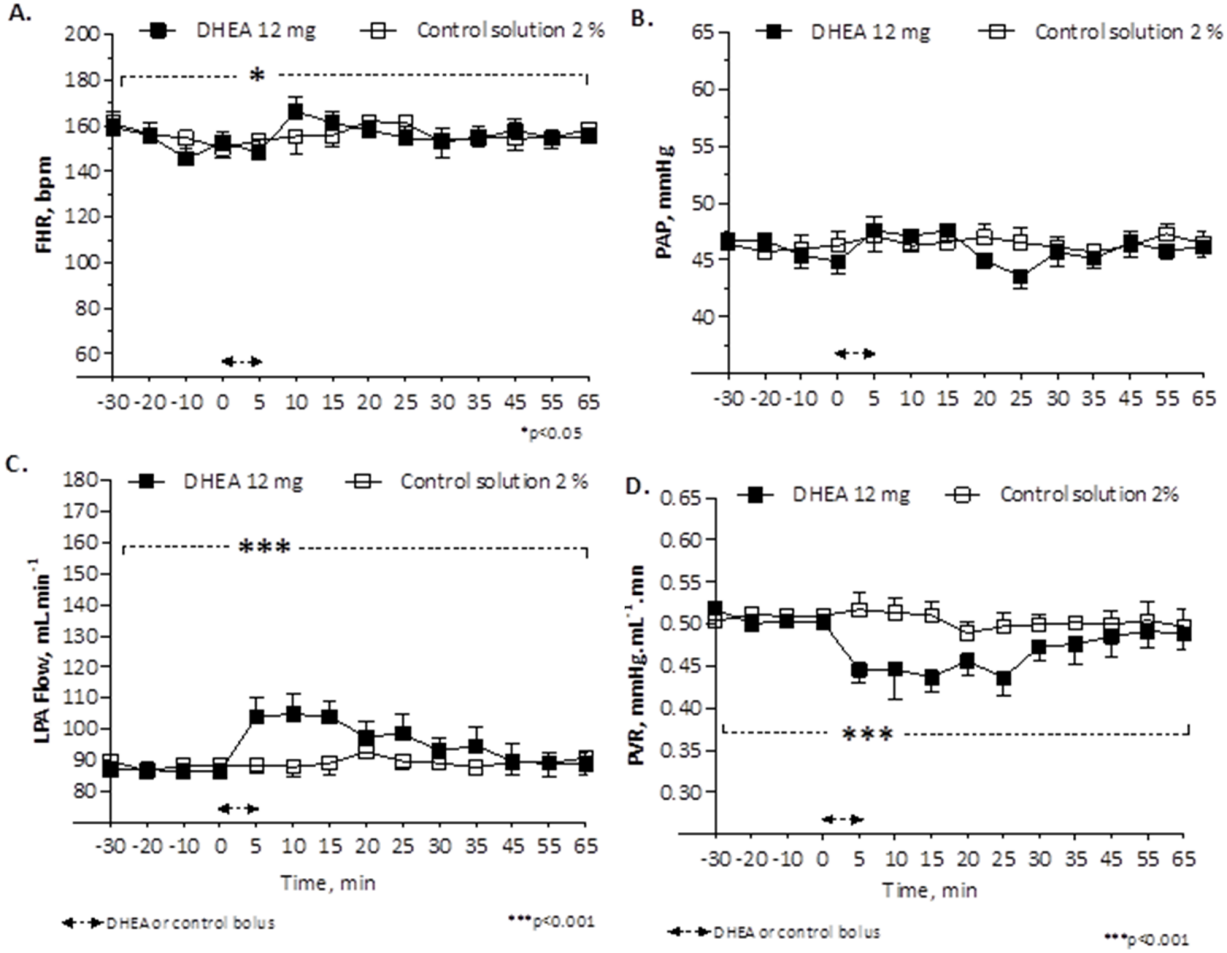
Change in fetal heart rate, FHR (A), pulmonary artery pressure, PAP (B), left pulmonary artery blood flow, LPA flow (C) and pulmonary vascular resistance, PVR (D) in response to bolus of 12 mg DHEA compared to bolus of control solution 2%. Values are means ±SEMs, n = 5, (DHEA 12 mg) and n = 5, (control 2%). *p<0.05 significant difference of FHR comparing baseline vs. 12 mg DHEA bolus and ***p<0.001 comparing baseline vs. 12 mg- DHEA bolus (one-way ANOVA) and comparing 12 mg- DHEA bolus vs. control 2% (Two-way ANOVA).

**Fig 3:**
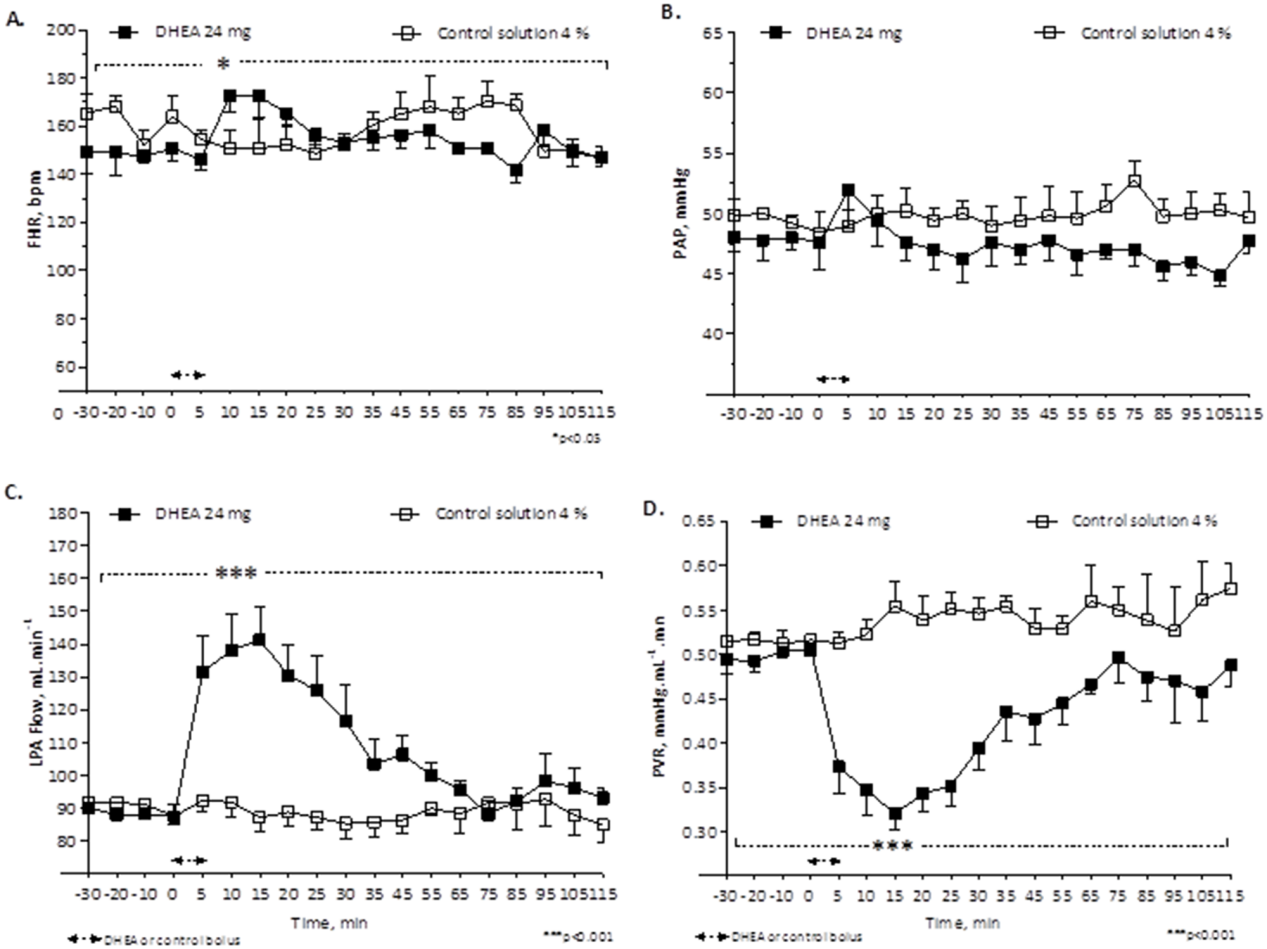
Change in fetal heart rate, FHR (A), pulmonary artery pressure, PAP (B), left pulmonary artery blood flow, LPA flow (C) and pulmonary vascular resistance, PVR (D) in response to bolus of 24 mg DHEA compared to bolus of control solution 4%. Values are means ±SEMs, n = 5, (DHEA 24 mg) and n = 4, (control 4%). *p<0.05 significant difference of FHR comparing baseline vs. 24 mg DHEA bolus ***p<0.001 comparing baseline vs. 24 mg- DHEA bolus (one-way ANOVA) and comparing 24 mg- DHEA bolus vs. control 4% (Two-way ANOVA).

A 5-min infusion of DHEA with 6 mg (n = 5, 7 experiments; Fig 1), 12 mg (n = 5, 7 experiments; Fig 2) or 24 mg (n = 5, 5 experiments; Fig 3) resulted in a decrease in PVR, with the largest decrease occurring 15 min after the bolus infusion (0.452 ±0.024 _(bolus 6 mg DHEA)_ vs. 0.511 ± 0.016 _(control solution 2%)_ mmHg·mL^-1^·min; 0.437 ±0.018 _(bolus 12 mg DHEA)_ vs. 0.511 ± 0.016 _(control solution 2%)_ mmHg·mL^-1^·min; and 0.321 ±0.018 _(bolus 24 mg DHEA)_ vs. 0.555 ± 0.027 _(control solution 4%)_ mmHg·mL^-1^·min, with a significant difference for 12 mg and 24 mg DHEA bolus [p<0.001]), associated to an increase in LPA blood flow (Figs 2C, 2D, 3C and 3D). This effect started 5 min after the bolus infusion, persisted into a maximum relaxation 15 min after the bolus infusion, and had a total duration of 35 min (Figs 1, 2 and 3). No effects on the fetal pulmonary or systemic circulation were observed with infusion of the control solution (Figs 1, 2 and 3).

**Fig 4:**
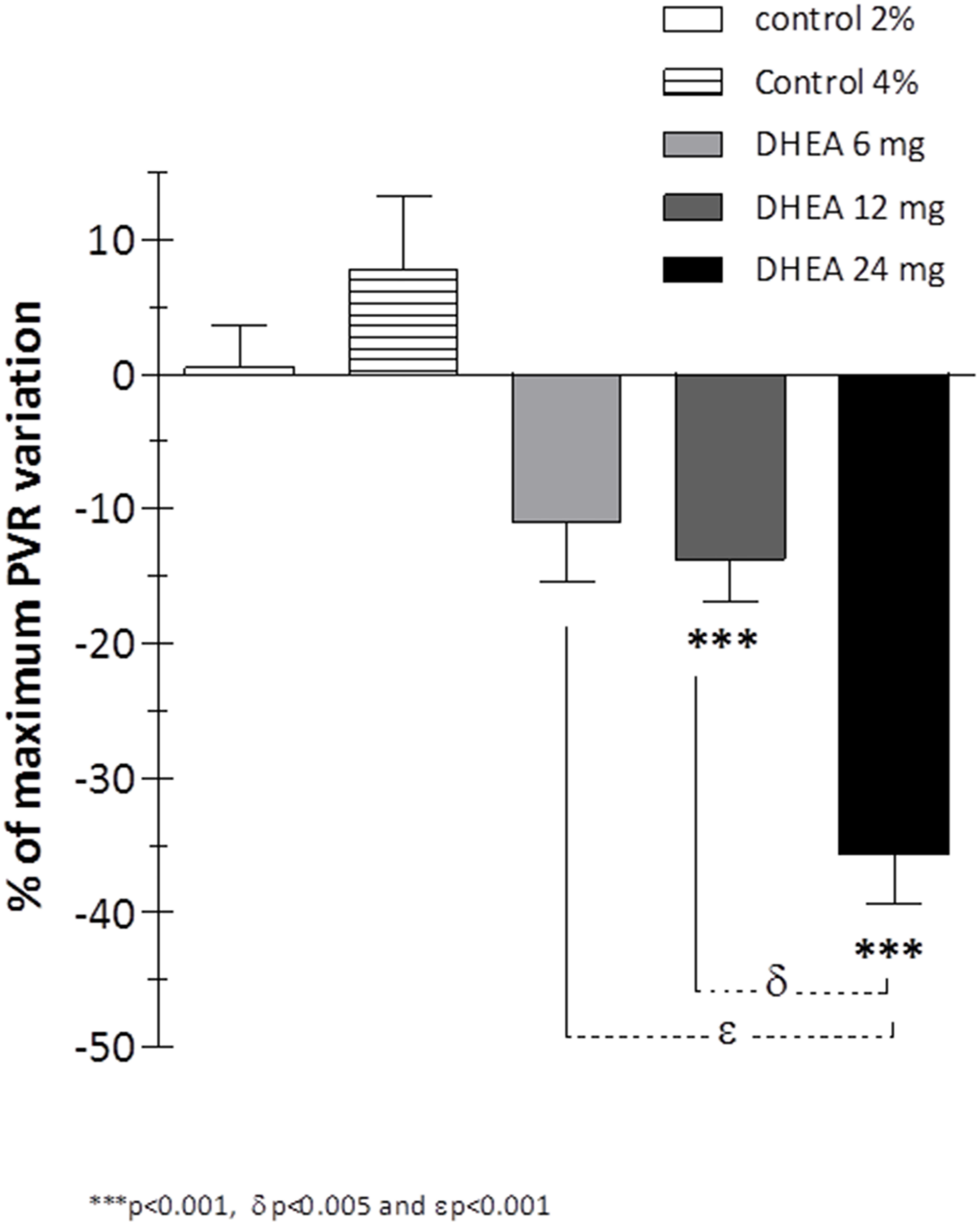
Groups-related changes in PVR with increasing doses of DHEA bolus infusion. Maximum percentage of variation of PVR with increasing doses of DHEA bolus infusion with significant difference in 24 mg DHEA group compared to 6 mg DHEA and to 12 mg DHEA groups (Two-way ANOVA).

The vasodilator effect increased gradually with the DHEA dose (6, 12 and 24 mg), showing a maximum percentage of variation of PVR at T15 min that was 11%, 13.8% and 35.7%, respectively (Fig 4). The vascular response that followed 24 mg bolus of DHEA was significantly greater than that following the 6 mg (p<0.001) or 12 mg (p<0.005) bolus of DHEA.

No significant difference was found between the blood gas measurements obtained 30 min before and 30 min after the bolus infusion of DHEA (p>0.05), regardless of the DHEA dose (6, 12 or 24 mg; Table 1).

**Table 1:** Comparisons of blood gases, heart rate, aortic and pulmonary arterial pressures 30 min before and 30 min after bolus infusion of DHEA (6, 12 and 24 mg).

Bolus	6 mg DHEA (n = 5)^b^			12 mg DHEA (n = 5)^b^			24 mg DHEA (n = 5)^b^		
Time	T-30	T30	*P*	T-30	T30	*P*	T-30	T30	*P*
**pH**	7.36±0.01	7.36±0.01	*NS*	7.36±0.01	7.37±0.01	*NS*	7.36±0.01	7.35±0.01	*NS*
**pCO**_**2**_^a^ **(mmHg)**	52±1.6	51±1.4	*NS*	51±1.4	50±1.2	*NS*	49±0.9	51±0.9	*NS*
**pO**_**2**_^a^ **(mmHg)**	15±1.4	15±1.5	*NS*	15±0.9	15±1	*NS*	15±1.4	16±1.9	*NS*
**Lactates (mM)**	1.6±0.1	1.3±0.1	*NS*	1.8±0.2	1.6±0.2	*NS*	1.8±0.2	1.7±0.2	*NS*
**SaO**_**2**_^a^ **(%)**	49±6	48±6	*NS*	46±4	46±3	*NS*	43±6	48±6	*NS*
**Hb**^a^ **(g/dL)**	11±0.3	11±0.3	*NS*	11±0.5	11±0.6	*NS*	12±0.6	12±0.5	*NS*
**PAo**^a^ **(mmHg)**	47±2.6	46±2.3	*NS*	44±1	44±1.3	*NS*	45±1.6	46±2	*NS*

^a^ pCO_2_: partial pressure of carbon dioxide; pO_2_: partial pressure of oxygen; SaO_2_: saturation in oxygen; Hb: hemoglobin; PAo: Aortic pressure

^b^ Results expressed in as mean± SEM; NS: not significant, p>0.05, n = number of independent observations

No systemic effect was observed during the bolus infusion of DHEA, except for a significant increase in fetal HR that occurred without changes in PAP (Figs 1, 2 and 3).

Blood levels of DHEA were significantly higher at T35 min than at T0 min in both the ewe (except for the 5-min infusion of 24 mg of DHEA) and fetus. However, increases in DHEA-S blood levels were significant only in the fetus. Table 2 presents the blood levels of DHEA and DHEA-S in ewes and fetuses following bolus infusion of DHEA. These blood levels were correlated with the increasing dose of DHEA infused. Higher blood levels of DHEA and DHEA-S were observed in fetuses with 24 mg DHEA than with 6 or 12 mg DHEA.

**Table 2:** Blood levels of DHEA and DHEA-S before and after bolus infusion of DHEA.

Bolus	6 mg DHEA (n = 7)^b^			12 mg DHEA (n = 7)^b^			24 mg DHEA (n = 5)^b^		
**DHEA**^a^ **(nmol/L)**	T0^a^	T35^a^	*p*	T0	T35	*p*	T0	T35	*p*
**Ewe**	0.19 ±0.02	0.8 ±0.3	*0*.*05*	0.24 ±0.05	**0.82 ±0.17**	***0*.*02***	0.26	0.85 ±0.14	*NS*
**Fetus**	0.17 ±0.02	**3.8 ±0.8**	***0*.*02***	0.43 ±0.06	**6.56 ±0.95**	***0*.*009***	0.4 ±0.04	**14.65 ±0.7**	***0*.*009***
**p**	NS	0.03		0.05	0.014		NS	0.05	
**DHEA-S**^a^ **(μmol/L)**	T0	T35	*p*	T0	T35	*p*	T0	T35	*p*
**Ewe**	<0.41	<0.41	*NS*	<0.41	<0.41	*NS*	<0.41	0.53 ±0.1	*NS*
**Fetus**	<0.41	**1.13 ±0.17**	***0*.*02***	<0.414	**1.34 ±0.26**	***0*.*036***	<0.41	**2.31 ±0.17**	***0*.*04***
***p***	*NS*	*0*.*03*		*NS*	*0*.*05*		*NS*	*NS*	

^a^ T0: time just before DHEA bolus; T35: time 30 min after DHEA bolus; DHEA-S: dehydroepiandrosterone sulfate

^b^ Results expressed as mean ±SEM; NS: not significant, p>0.05, n = number of independent observations

### Expt. 2: Infusion of 30 mg LNA before the bolus infusion of 12 mg and 24 mg of DHEA inhibited the vasodilator effect on fetal pulmonary circulation

**Fig 5:**
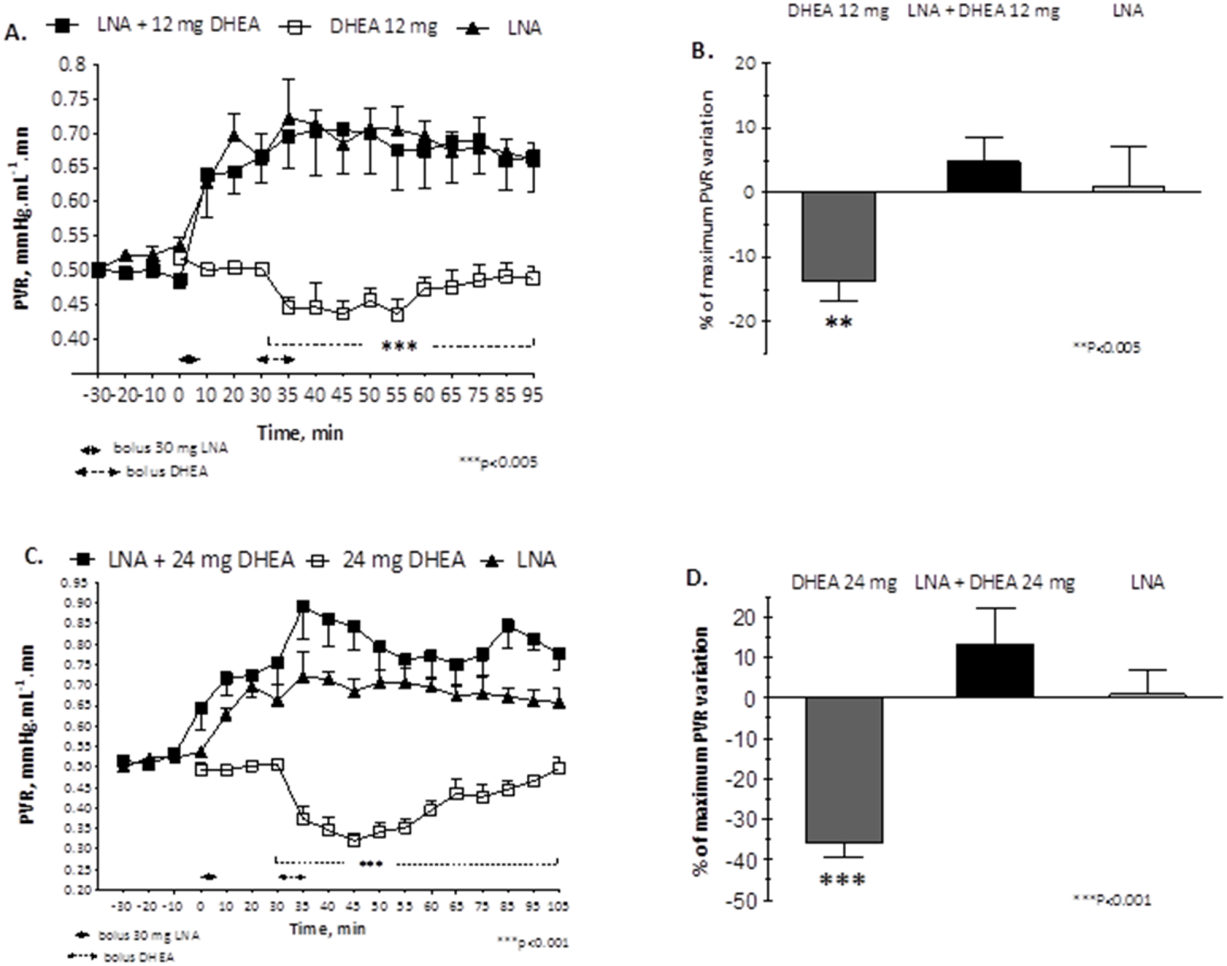
Time-related changes in PVR before and 15 min after DHEA bolus infusion, 30 min after 30 mg LNA bolus infusion. (A) comparison of PVR between 30 mg LNA infusion (n = 5), DHEA 12 mg infusion (n = 5) and LNA+DHEA 12 mg infusion (n = 5); (B) maximum percentage of PVR with 30 mg LNA, or DHEA 12 mg bolus infusion with and without 30 mg LNA pre-treatment; (C) comparison of PVR between 30 mg LNA infusion (n = 5), DHEA 24 mg infusion(n = 5) and LNA+DHEA 24 mg infusion (n = 4); (D) maximum percentage of PVR with 30 mg LNA, or DHEA 24 mg bolus infusion with and without 30 mg LNA pre-treatment.

There was a trend of an increase, rather than a decrease, in PVR after a 5-min infusion of 12 mg DHEA (n = 5, 6 experiments (Fig 5A) or 24 mg DHEA (n = 4, 5 experiments; Fig 5C) when the fetus was pre-treated with a 30 mg LNA infusion. The maximum variations in PVR were + 4.81% (12 mg of DHEA, p = 0.65) and +13.12% (24 mg of DHEA, p = 0.38), but this difference was not significant (Fig 5B and 5D). No effects on the systemic circulation (HR, PAo, PAP) or significant differences among blood gases before and after LNA-DHEA infusion were observed (data not shown). Change in PVR to 12 mg and to 24 mg DHEA after pre-treatment with LNA were not significantly different from change in PVR to LNA alone (p = 0.2081, Two-way ANOVA; Fig 5A and 5C).

Pre-treatment with 30 mg of LNA infusion significantly inhibited the vasodilator effect on PVR previously observed with DHEA infusion (p<0.005 for 12 mg of DHEA compared to LNA+DHEA 12 mg, and p<0.001 for 24 mg of DHEA compared to LNA+DHEA 24 mg; Two-way ANOVA; Fig 5B and 5D).

## Discussion

Refractory hypoxemia may persist in PPHN despite traditional therapeutic approaches. In this study, we demonstrate a vasodilator dose-dependent effect of DHEA on fetal pulmonary circulation, with an increase in LPA blood flow and a significant decrease of PVR.

For the first time, we demonstrate a vasodilator effect of DHEA on fetal pulmonary circulation, with an increase in LPA blood flow and a significant decrease in PVR (11 to 36%) that were related to the dose of DHEA (6 to 24 mg) infused.

This is the first study that we are aware of to observe this vasodilator effect in the pulmonary circulation in animals with PPHN physiology. Other studies have investigated the effects of DHEA on the pulmonary vasculature in chronic hypoxic PH states using oral treatment (DHEA mixed with the diet of rats) [17–20, 27–29]. Bonnet et al [18] administered DHEA in chronic hypoxia in rats and found a similar vasodilator dose-dependent effect on the pulmonary vasculature as we did in this study. Dumas de la Roque et al. [27] demonstrated both a preventive and curative effects of DHEA with oral treatment (30 mg.kg-1 of DHEA) in a rat pup-model with hypoxic PH. Potential mechanisms of action related to calcium channels are discussed below [30,31]. In addition to the vasodilator effect in the pulmonary circulation, there has been recent literature that suggests different and opposing actions of the affect DHEA on the cardiovascular system (coronary and systemic circulation) [32–34]. Molinari et al. observed an increase in myocardial contraction and coronary perfusion pressure following DHEA administration in prepubertal anesthetized pigs. In this experiment, the authors demonstrated a rapid vasoconstrictor effect of DHEA on the cardiac system when pigs were injected with a direct intravenous bolus followed by a prolonged infusion; however they also found a significant decrease in coronary blood flow, but with a stable HR and normal aortic blood pressure [32]. In our study, we observed a significant increase in HR in fetal lambs during DHEA perfusion that that occurred without changes in aortic or pulmonary arterial pressures. The short duration of action observed in our work may also be explained by a placental removal of DHEA. Indeed, the maternal blood levels of DHEA significantly increased after DHEA was injected into the fetus. Thus, our results tended to show a passage of DHEA through the placenta, from the fetus to the mother. In addition, Molinari et al. found a very rapid vasoconstrictor effect on the cardiac system beginning at three minutes and concluding five minutes after the injection [33]. In the present study, 6 to 24 mg (1.6 to 6.5 mg·kg^-1^) of DHEA was administered to fetal lambs, which did not exceed the doses of DHEA given for treatment of PPH in rat models (adult and pups: 9 to 30 mg·kg^-1^ per os) [17,18,27] or in adult humans (200 mg per os corresponding to 2.5 to 4 mg·kg^-1^) with chronic obstructive pulmonary disease [27]. Regarding the blood levels of DHEA-S, the doses (1.13 ±0.17 μM [6 mg DHEA] to 2.31± 0.17 μM [24 mg DHEA]) reported in fetal lambs were similar to the physiological levels of DHEA-S reported for the human fetus during gestation (between 2 and 6 μM, decreasing with gestational age) [12]. Moreover, the DHEA doses in our study (with conversion [ng·mL^-1^/3.47]: 3.80±0.8 nmol·L^-1^ [6 mg DHEA] to 14.65 ±0.7 nmol·L^-1^ [24 mg DHEA]) were lower than the mean serum concentration of free DHEA reported in humans (measured in the morning before taking DHEA) [27] at baseline levels (11.2 [9.6–12.3] nmol·L^-1^) and after 3 months of DHEA treatment (23.4 [19.7–38.4] nmol·L^-1^). Thus, the doses of DHEA used for medical treatments and the blood concentrations of DHEA/DHEA-S measured in the fetus in the present study are similar to those reported in experimental or clinical studies and suggest the potential use of DHEA in neonatal care. It has also been showed that endogenous levels of DHEA increased in women participating in both resistance and endurance exercises [35,36]. We could therefore hypothesize that exercise during pregnancy might improve pulmonary circulation at birth by the increase of endogenous levels of DHEA in both mother and fetus, but this has not yet been properly tested.

To understand the mechanism of action of DHEA, we tested the effect of DHEA after pre-treatment with LNA, an inhibitor of the NO pathway. Our results suggest that NOS inhibition prevents DHEA-mediated pulmonary vasodilation.

At the cellular level, DHEA affects both endothelial and smooth muscle cells by activating or inhibiting many different signaling pathways through binding membrane-associated receptors or ionic channels: activation of eNOS and production of NO, activation of Big calcium-sensitive potassium channels (BKCa), inhibition of T-type voltage operated calcium (VOC) channels, inhibition of Rho A and ROCK pathway, and up-regulation of soluble guanylate cyclase protein and cyclic GMP [13,15,29–31,34]. In vitro, DHEA treatment of pulmonary smooth muscle cells, including those obtained from hypoxic pulmonary vessels in the ferret lung and cultured explants of human pulmonary arteries, showed a complete reversibility of hypoxic pulmonary vasoconstriction [22,23]. The mechanism described includes passage through the opening of KCa, and a change in the redox potential of smooth muscle cells in the pulmonary artery [18,22,23]. In our study, pre-treatment with LNA inhibited the vasodilator action of DHEA through a vasoconstrictor effect. Our results therefore suggest a vasodilator mechanism of action for DHEA that is mediated by NO. Some authors have suggested that vascular action of DHEA and DHEA is not mediated by their androgen or estrogen receptors but that DHEA acts directly on endothelial cells via a cell surface receptor coupled to a G-protein to stimulate NOS activation [23,37–39]. Other studies indicate that DHEA might inhibit the phosphatidyl inositol 3-Kinase (PIK3)/Akt signaling pathway via membrane-associated DHEA receptors [40]. Specific receptors of DHEA are mentioned but their molecular structures are not yet described. Recently, it has been suggested that DHEA improves cardiac function via the activation of sigma-1 receptor which is involved in Akt/eNOS signaling pathway in ovariectomized rats [41]. This receptor might represent a new target of DHEA in cardiovascular diseases.

This study had some limitations. In pilot study, we observed a precipitation of DHEA powder and obstruction of fetal catheter occurring during slow intravenous infusion and thus, explaining why we used DHEA with 5-min bolus infusion.

DHEA might have gender-dependent effect and because there is no sex ratio in PPHN, the gender of the lamb fetuses was not collected and no sex grouping was done. It could represent another limitation of this study.

To explain the pulmonary vasodilator effect of DHEA, we investigated the role of DHEA on the NO signalling pathway. We cannot exclude that other mechanisms may explain the vascular response to DHEA, including changes in prostacyclin signalling pathway. However, to the best of our knowledge, no data exist regarding the effects of DHEA on endothelial prostacyclin pathway.

In conclusion, the present study is the first to report a potent vasodilator effect on fetal pulmonary circulation, and this result has motivated us to consider whether DHEA is a potential treatment for PPH in neonatal care.
